# Supplementary material for: MetaRibo-Seq measures translation in microbiomes
Source: Nat Commun. 2020 Jun 29;11:3268. doi: 10.1038/s41467-020-17081-z (PMC7324362; doi:10.1038/s41467-020-17081-z)
Supplement: Supplementary file 10 — Supplementary Data 7 [file 41467_2020_17081_MOESM10_ESM.zip › File2/Confidence_High_Taxonomy/155800_out.krona.html]

Javascript must be enabled to view this page.

members
magnitude
magnitudeUnassigned
count
unassigned
taxon
rank

155800\_out

6

6
2
superkingdom

1239
phylum
5

5
class
186801

5
186802
order

family
31979
5

5
1485
genus

species
1262820
5

SRS012273\_contig\_number\_16863SRS022071\_contig\_number\_30595SRS063985\_contig\_number\_22130SRS148159\_contig\_number\_37590SRS971275\_contig\_number\_5119

1
phylum
976

class
200643
1

1
order
171549

815
family
1

genus
816
1


SRS011239\_contig\_number\_12748
1
species
585544
